# Positive inotropic effects of serotonin in atrial EHT: further proof for an atrial phenotype?

**DOI:** 10.1007/s00210-025-04619-5

**Published:** 2025-10-28

**Authors:** Muhammed Sönmez, Margaret Nandudu, Paul Brunnbauer, Carl Schulz, Birgit Klampe, Thomas Schulze, Arne Hansen, Thomas Eschenhagen, Torsten Christ

**Affiliations:** 1 University Medical Center Hamburg-Eppendorf, Institute of Experimental Pharmacology and Toxicology, Martinistraße 52, 20246 Hamburg, Germany; 2 Department of Cardiology, University Medical Center Hamburg-Eppendorf, Martinistreet 52, 20251, Hamburg, Germany; 3 DZHK (German Centre for Cardiovascular Research), Partner Site Hamburg/, Kiel/Lübeck, Hamburg, Germany

**Keywords:** Serotonin (5-HT), 5-HT4 receptor, Human-induced pluripotent stem cell-derived cardiomyocytes (hiPSC-CM), Engineered heart tissue (EHT), Action potential, Calcium current

## Abstract

Human-induced pluripotent stem cell-derived cardiomyocytes (hiPSC-CM) cultured in the presence of retinoic acid were proposed as a model for human atrium. Since in the human heart positive inotropic effects of serotonin (5-HT) are restricted to the atrium, we investigated whether the presence of 5-HT responses may distinguish between hiPSC-atrial and ventricular CM. Nonfiltered retinoic acid (1 µM) was used to induce an atrial phenotype (hiPSC-aCM) in two different hiPSC lines. Untreated hiPSC-CM were used as controls (ventricular). We measured 5-HT4 receptor (*HTR4*) expression and 5-HT effects on Ca^2+^ currents (I_Ca_), force, and action potential in engineered heart tissue (EHT). *HTR4* transcript abundance was five times higher in atrial EHT. 5-HT (100 µM) increased I_Ca_ from 5.3 ± 0.8 to 8.1 ± 1.1 pA/pF, prolonged APD_20_ from 8.1 ± 1.2 to 12.0 ± 1.2 ms, and shortened APD_90_ from 142.3 ± 11.3 to 117.0 ± 6.6 ms in hiPSC-aCM, but not in hiPSC-vCM. 5-HT increased force in atrial EHTs from 0.06 ± 0.01 to 0.10 ± 0.01 mN but not in vEHT. Spontaneous beating rate per minute was increased by 5-HT in aEHT from 151.3 ± 7.5 to 173.7 ± 4.1 and in vEHT from 43.1 ± 4.2 to. 61.3 ± 3.4. Both EHTs contain functional 5-HT receptors, but 5-HT increases I_Ca_ and force only in aEHT, similar to the human heart. In vEHT, 5-HT’s positive chronotropic effect in the absence of I_Ca_ stimulation and inotropic response suggests activation of funny channels. Our findings demonstrate that aEHT shares the typical atrial-selective regulation of I_Ca_ and force by 5-HT, thus making aEHT a promising model to study 5-HT_4_ receptors in the human heart.

**Supplementary Information:**

The online version contains supplementary material available at 10.1007/s00210-025-04619-5.

## Introduction

Human-induced pluripotent stem cell-derived atrial cardiomyocytes (hiPSC-aCM) have opened the possibility of studying AF gene-variant effects in an experimental system with a human genetic background. More than 350 genes have a significant association with AF risk (Roselli et al. 2025). However, it is open to which extent atrial hiPSC-CM resemble the situation in the human adult atrium.

The human ventricle and atrium differ substantially in the expression of some transcription factors, myofilaments, and ion channels (Ellinghaus et al. 2005; Gaborit et al. 2007; Lemme et al. 2018) and action potential (AP) shape (Trautwein et al. 1962). Consequently, much effort has been spent to recapitulate atrial AP shape in hiPSC-aCM (Devalla et al. 2015; Lee et al. 2017; Lemme et al. 2018; Schulz et al. 2023). In this study, we focus on a peculiarity of the human atrium.

In the human heart, serotonin (5-HT) increases membrane calcium currents (I_Ca_), in atrial (Ouadid et al. 1992) but not in ventricular (Jahnel et al. 1992) cardiomyocytes. In line with this, 5-HT increases the force of the human atrium but not in the human ventricle (Jahnel et al. 1992; Kaumann et al. 1990). Furthermore, 5-HT induces arrhythmias in the human atrium under in vitro conditions (Kaumann and Sanders 1994; Pau et al. 2007). Importantly, in the context of translational research, the 5-HT-induced increase in cAMP, inotropy, and arrhythmogenesis (Berk et al. 2016; Christ et al. 2014; Dolce et al. 2020) is blunted in human chronic AF (Dolce et al. 2020). Therefore, 5-HT response on hiPSC-CM could be useful in studying disease-related atrial remodelling.

Thus, we felt it was worth investigating whether hiPSC-aCM may reproduce physiological effects of 5-HT seen in human atrium.

## Methods

### Engineered heart tissue from hiPSC-aCM

#### Atrial differentiation of hiPSC and generation of atrial EHT

The investigation of hiPSC-CM conforms to the principles outlined by the Declaration of Helsinki, with skin fibroblasts being obtained with the informed consent of the donors. All procedures involving the generation and analysis of hiPSC lines were approved by the local ethics committee in Hamburg (Az PV4798, 28.10.2014). With regard to the protocols, in-house hiPSC expansion was performed: hiPSC atrial cardiomyocyte (aCM) with non-filtered 1 µM retinoic acid or without (hiPSC-vCM) were differentiated and EHTs were generated as recently described (Breckwoldt et al. 2017). Initially, embryoid bodies (EBs) were generated from expanded hiPSCs using spinner flasks and stirred suspension. Mesodermal induction was executed by growth factor cocktail (BMP-4 10 ng/ml, activin A 3 ng/ml, R&D Systems, Minneapolis, USA; bFGF 5 ng/ml, Miltenyi Biotec, Cologne, Germany) for 3 days and the cardiac differentiation was induced by a WNT signal inhibitor XAV 939 (1 µM, Tocris, Wiesbaden-Nordenstadt, Germany).

A total of 51 atrial EHT (33 from 3 batches of ERC001, 18 from 3 batches of ERC018-hiPSC-aCMs) and 40 ventricular EHT (32 from 3 batches of ERC001, 8 from 3 batches of ERC018 hiPSC-vCM) were utilized in this study. For the generation of one EHT, we used 1.0 × 10^6^ hiPSC-CMs. The fibrin gel matrix was prepared by combining hiPSC-CMs, fibrinogen (Sigma F4753, St. Louis, USA), and thrombin (100 U/L, T7513, St. Louis, USA), which were added to agarose (1%) casting molds with silicone posts, as previously described by Breckwoldt et al. (). EHTs were cultured for 30 days under identical conditions at 37 °C in a 7% CO_2_ and 40% O_2_ humidified cell culture incubator with a medium consisting of DMEM (Biochrom, Berlin, Germany), 10% heat-inactivated horse serum (Gibco, Paisley, Scotland), 1% penicillin–streptomycin (Gibco, Paisley, Scotland), insulin (10 µg/mL; Sigma, St. Louis, MO, USA), and aprotinin (33 µg/mL; Sigma, St. Louis, MO, USA).

### Expression analysis by RNA sequencing

The expression analysis by RNA sequencing was performed by the Core Facility Genomics in Muenster (Medical Faculty, Muenster, Germany). Total RNA was extracted from frozen EHTs using the RNeasy Mini Kit (Qiagen). The high-quality total RNA (Agilent TapeStation 4200, RIN value check) was then enriched for poly(A) tailed RNA using the NEBNext Poly(A) magnetic isolation module (NEB) followed by NEBNext Ultra II directional RNA Library Preparation (NEB). The library was then subjected to quality control (Agilent TapeStation 4200), quantification (NEBNext Library Quant for Illumina, NEB), and an equimolar pool was sequenced in a single read mode, 72 cycles on a NextSeq 2000 system (Illumina).

### Current measurements

To measure ion currents in hiPSC-vCM or hiPSC-aCM, EHTs were dissociated (Breckwoldt et al. 2017) using collagenase II (200 U/ml, 1 mg/ml, Worthington, NJ, USA) for a duration of 3.5 h at temperature of 37 °C. Isolated hiPSC-CMs were plated on gelatine-coated (0.1%) glass coverslips (12 mm diameter; Carl Roth GmbH + Co, Karlsruhe, Germany) and stayed in culture for 24–48 h to maintain adherence under superfusion in the recording chamber during patch clamp measurements.

Ion currents were recorded at 37 °C in the whole-cell configuration using an Axopatch 200B amplifier (Axon Instruments, Foster City, CA, USA). ISO2 software (MFK, Niedernhausen, Germany) was used for data acquisition and analysis. Heat-polished pipettes were pulled from borosilicate filamented glass with an external diameter of 1.5 mm and internal diameter of 0.87 mm (HILG1103227; Hilgenberg, Malsfeld, Germany) utilizing a DPZ-Universal puller (Zeitz Instruments, Munich, Germany). Tip resistances were measured to be in the range of 2.5–5 MΩ, while seal resistances were in the range of 3–6 GΩ. Cell capacitance (C_m_) was calculated from steady-state current during depolarizing ramp pulses (1V/1s) from − 40 to − 35 mV. The cells were investigated in a small perfusion chamber placed on the stage of an inverse microscope. I_Ca_ were stimulated by applying test pulses from − 80 to + 10 mV (200 ms) at 0.5 Hz. The amplitude of the current was calculated as the difference between the peak and the late inward current. Extracellular Ca^2+^ concentration was 2 mM. K^+^ currents were blocked by Cs^+^ and tetraethylammonium chloride in the bath solution. The experiments were performed as previously mentioned (Christ et al. 2014) with the following Na^+^-free bath solution (in mM): tetraethylammonium chloride 120, CsCl 10, HEPES 10, CaCl_2_ 2, MgCl_2_ 1, and glucose 20 (pH 7.4, adjusted with CsOH). The pipette solution (pH 7.2, adjusted with CsOH) included (in mM) cesium methanesulfonate 90, CsCl 20, HEPES 10, Mg-ATP 4, Tris-GTP 0.4, EGTA 10, and CaCl_2_.

### Action potential measurement

Action potentials in intact atrial trabeculae and atrial EHT were recorded with standard sharp microelectrodes pulled from the same mentioned above glass capillaries (Lemoine et al. 2017). The tip resistance of these microelectrodes ranged from 25 to 55 MΩ when filled with 2 M KCl. Tissues were continuously perfused with Tyrode’s solution (in mM): NaCl 127, KCl 5.4, MgCl_2_ 1.05, CaCl_2_ 1.8, glucose 10, NaHCO_3_ 22, and NaHPO_4_ 0.42 and balanced with O_2_-CO_2_ [95:5] at 36 °C, pH 7.4. The atrial EHTs were removed from the 24-well EHT culture plate into the AP measuring chamber and were fixed with needles for AP recording. The signals were amplified by a BA-1s npi amplifier (npi electronic GmbH, Tamm, Germany). APs were recorded and analyzed off line using the Lab-Chart software (version 8, AD Instruments Pty Ltd., Castle Hill, NSW, Australia). A single concentration (100 µM) of 5-HT (Sigma, St. Louis, USA) or norepinephrine (NE, Sigma, St. Louis, USA) was used to measure maximum effects of both agonists on AP shape. Additionally, effects on spontaneous beating rate were measured using a single concentration of ivabradine (300 nM; Sigma, St. Louis, USA).

The AP parameters were analyzed as action potential amplitude (APA) and maximum diastolic potential (MDP), with AP duration at 20, 50, and 90% of repolarization (APD_20_, APD_50_, APD_90_, respectively). The plateau potential (V_Plateau_) was defined as the mean absolute membrane potential in a 5-ms window starting from 20% of APD_90_, and maximum upstroke velocity (V_max_) as previously (Pecha et al. 2023). The APs were recorded and analyzed using the Lab-Chart software (AD Instruments Pty Ltd., Castle Hill, NSW, Australia). Effects were measured after 2–3 min adding the 5-HT or NE and 30 min after ivabradine. To calculate the AP parameters, data from 15 consecutive APs were averaged.

### Force measurement

Video-optical recording methodology as previously described, was used to measure force (Hansen et al. 2010; Saleem et al. 2020). In summary, a video camera was placed above the recording chamber; deflection of the silicone posts was recorded with a specific software (CTMV, Pforzheim, Germany). The contraction peaks were analyzed in terms of frequency and force and were measured at 37 °C.

EHTs were incubated with 0.6 mM Ca^2+^ for 30–45 min to facilitate detection of positive inotropic effects (Mannhardt et al. 2017). Next, 5-HT or NE was added cumulatively in half log steps from 10^−9^ to 10^−4^ M. Finally, Ca^2+^ concentration was increased to 1.8 mM to reach the maximum force response.

## Results

### *HiPSC-aCM express genes encoding for 5-HT*_*4*_* receptors more than hiPSC-vCM*

Transcript levels of 5-HT_4_ in the right ventricle amounts to 41% of that in the right atrium (Gergs et al. 2009). Thus, we analyzed by bulk RNA sequencing whether this holds true for ventricular and atrial EHTs generated from hiPSC-vCM and hiPSC-aCM, respectively. We found mRNA abundance of 5-HT_4_ receptor in hiPSC-aCM fivefold higher than hiPSC-vCM (Figure S1).

### 5-HT increased I_Ca_ in hiPSC-aCM but not hiPSC-vCM

**Fig. 1:**
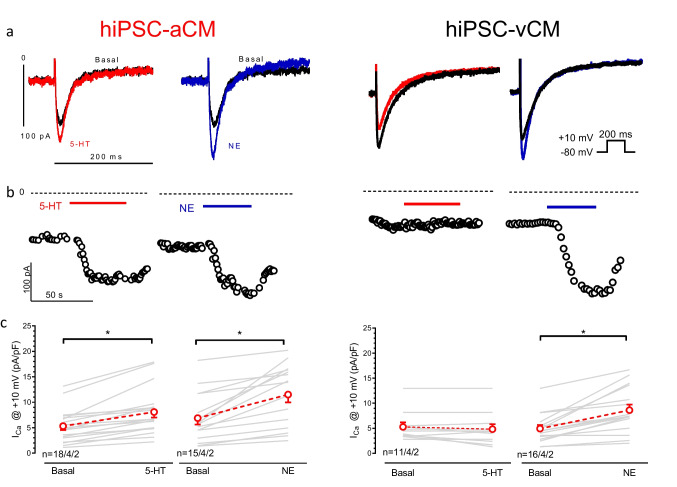
**5-HT increased I**_**Ca**_ **in hiPSC-aCM but not hiPSC-vCM**. **a** Superimposed original traces of calcium currents (I_Ca_) basal and exposed 5-HT (100 µM, red) or NE (100 µM, blue) in hiPSC-aCM (left) and in hiPSC-vCM (right). Pulse protocol given as inset. **b** Respective time courses of the current. **c** Summary of data. Mean ± SEM of I_Ca_ before (basal) and after adding 5-HT or NE in hiPS-aCM (left) or in hiPSC-vCM. (right) *p < 0.05, nested t-test, n/n/n indicates hiPSC-CM/EHT/Cell line

5-HT increases I_Ca_ through 5-HT_4_ receptor in human atrial CM (Ouadid et al. 1992), but not in ventricular CM (Jahnel et al. 1992; Uzun et al. 2016). In order to investigate whether this difference also holds true for hiPSC-CM, we measured I_Ca_ responses to a single high concentration (100 µM) of 5-HT in hiPSC-aCM and hiPSC-vCM. I_Ca_ density under basal conditions (in the absence of 5-HT or NE) was similar in hiPSC-aCM and hiPSC-vCM (Table 1). Within 30 s of exposure to 5-HT, I_Ca_ increased in hiPSC-aCM from 5.3 ± 0.8 to 8.1 ± 1.1 pA/pF (*p* < 0.05, nested *t*-test, *n* = 18/4/2; hiPSC-aCMs/EHTs/cell lines, Fig. 1). For comparison, 100 µM NE increased I_Ca_ from 6.9 ± 1.3 to 11.6 ± 1.5 pA/pF in hiPSC-aCM (*p* < 0.05, *n* = 15/4/2) Thus, the response to NE in hiPSC-aCM was significantly larger than to 5-HT (*p* < 0.05, 4.8 ± 0.9 to 2.7 ± 0.5 pA/pF, Fig. 1, S2). 5-HT did not increase I_Ca_ in a single hiPSC-vCM recording, while NE, as expected, increased I_Ca_ from 4.9 ± 0.7 to 8.6 ± 1.1 pA/pF (*p* < 0.05, *n* = 16/4/2, Fig. 1, S2) as a confirmation of proper cAMP/PKA signalling in hiPSC-vCM.

**Table 1:** Values of calcium current in response to exposure to 100 µM 5-HT or 100 µM NE

	Basal	100 µM 5-HT	Basal	100 µM NE
hiPSC-aCM (pA/pF)	5.3 ± 0.8 (18/4/2)	8.1 ± 1.1* (18/4/2)	6.9 ± 1.3 (15/4/2)	11.6 ± 1.5* (13/4/2)
hiPSC-vCM (pA/pF)	5.3 ± 0.9 (11/4/2)	4.8 ± 1.0 (11/4/2)	4.9 ± 0.7 (16/4/2)	8.6 ± 1.1* (16/4/2)

Summary of calcium current (I_Ca_) under basal conditions, mean ± SEM, n/n/n indicates number of hiPSC-CMs (hiPSC-atrial or ventricular CM)/number of EHTs/cell lines, * indicates *P*-value < 0.05. basal vs. 5-HT or NE (nested *t*-test)

### In aEHT but not in vEHT, 5-HT prolonged plateau phase of AP

**Fig. 2:**
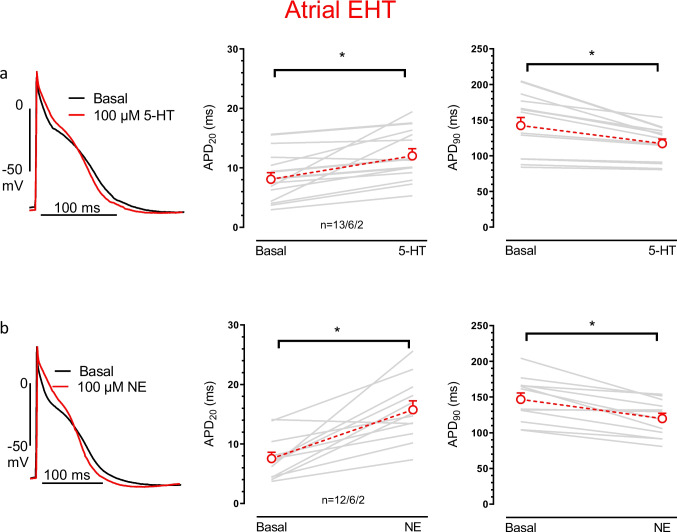
**5-HT enlarged plateau duration in atrial EHT**. Original action potential (AP) traces and data set of AP duration. AP recorded before (basal) and after adding 100 µM 5-HT (**a**, left) or 100 µM NE (**b**, left). Summary of action potential duration at % 20 repolarization (APD_20,_ central) or action potential duration at % 90 repolarization (APD_90_, right) values before (basal) and after adding 5-HT or NE. Mean ± SEM. *p < 0.05, nested *t*-test, n/n/n indicates number of EHT/batch/cell line

In human atrial CM, 5-HT increases APD_50_ (Pau et al. 2007). Therefore, we measured the effect of 5-HT (100 µM) on APs in atrial EHT (Table 2). 5-HT prolonged APD_20_ from 8.1 ± 1.2 to 12.0 ± 1.2 ms (*p* < 0.05, nested *t*-test, *n* = 13/6/2), and NE increased APD_20_ from 7.6 ± 1.0 to 15.8 ± 1.5 ms (*p* < 0.05, *n* = 12/6/2, Fig. 2, S3, no difference between two cell lines). Thus, NE had a much bigger effect on APD_20_ than 5-HT (delta 8.2 ± 1.5 vs. 3.9 ± 1.1 ms, *p* < 0.05, Table 2). APD_90_ was shortened with 5-HT (from 142.3 ± 11.3 to 117.0 ± 6.6 ms (*p* < 0.05, n = 13/3/2) and NE (from 147.5 ± 8.4 to 119.4 ± 6.8 ms, *p* < 0.05, nested *t*-test, Fig. 2, S3). The effect size on APD_90_ did not differ significantly between NE and 5-HT (shortening of 26.8 ± 5.5 vs. 25.3 ± 5.5 ms, Table 2).

**Table 2:** Values of action potential responses of atrial-EHTs exposed to 5-HT or NE

	Basal (13/3/2)	100 µM 5-HT (13/3/2)	Basal (12/3/2)	100 µM NE (12/3/2)
MDP (mV)	− 66.4 ± 2.4	− 67.4 ± 1.9	− 67.0 ± 2.6	− 65.6 ± 3.2
APA (mV)	88.8 ± 3.5	87.1 ± 2.8	91.1 ± 4.0	83.1 ± 5.2
V_max_ (V/s)	209.7 ± 31.9	179.9 ± 38.2	256.2 ± 38.7	200.6 ± 40.8
APD_20_ (ms)	8.1 ± 1.1	12.0 ± 1.2*	7.5 ± 1.0	15.8 ± 1.5*
APD_50_ (ms)	39.0 ± 2.2	48.4 ± 2.2*	41.6 ± 2.7	54.3 ± 2.4*
APD_90_ (ms)	142.3 ± 11.4	117.0 ± 6.7	146.6 ± 9.1	121.9 ± 6.6*
V_Plateau_	− 9.0 ± 1.8	− 9.7 ± 1.9	− 7.9 ± 1.8	− 7.7 ± 3.8
Beating rate (bpm)	188.3 ± 19.0	219.0 ± 13.0*	167.1 ± 11.8	221.3 ± 14.5*

Summary of AP characteristics under basal conditions, 100 µM 5-HT or 100 µM NE. MDP: maximum diastolic potential, APA: action potential amplitude, V_max_: maximum upstroke velocity, APD_20,50,90_: action potential duration % 20,50,90 repolarization. V_plateau_: plateau phase voltage, Beating rate: spontaneous beating rate per minute. Mean ± SEM, n/n/n indicates number of EHT/number of batches in case of atrial EHTs, * indicates *P*-value < 0.05. basal vs. 5-HT or NE (nested *t*-test)

### 5-HT showed an inotropic effect in atrial EHT but not ventricular EHT

In the human heart, 5-HT increases force in the atrium but not in the ventricle (Jahnel et al. 1992). To evaluate whether hiPSC-CM share this response pattern, we measured force responses to cumulatively increasing concentrations of 5-HT from 10^−9^to 10^−4^ M in both atrial and ventricular EHT. In order to compare maximum effect sizes, some EHTs were exposed to NE only. The basal force (before adding 5-HT or NE) was not different between groups exposed to 5-HT or NE.

**Fig. 3:**
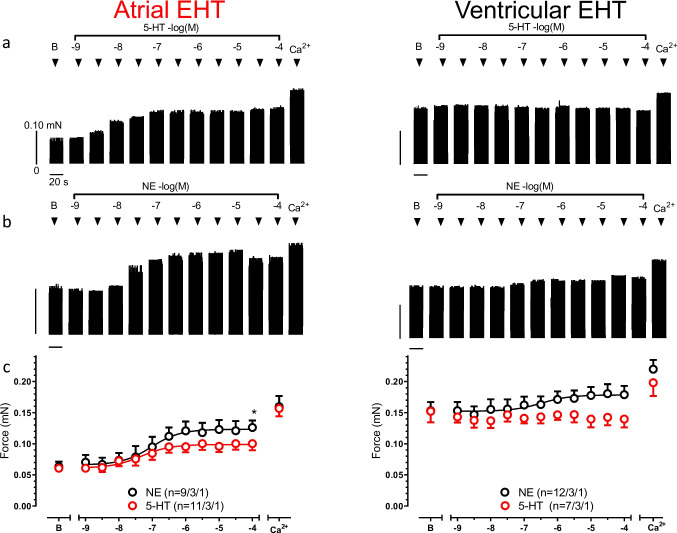
**5-HT increased the force of contraction in atrial EHT but not ventricular EHT**. Respective time courses of contraction basal at 0.6 mM Ca^2+^ as “**B**” and at the increasing concentration of 5-HT (**a**) or NE (**b**) (from 10^−9^ to 10^−4^ M) in atrial (left) and ventricular EHT (right). Data set of contractions at the increasing 5-HT and NE concentrations in atrial (left) and ventricular (right) EHT (**c**). Basal indicates as “**B”** at 0.6 mM Ca^2+^ and maximum effect at 1.8 mM Ca^2+^ “**Ca**.^**2+**^**”**. Mean ± SEM, n/n/n indicates number of EHT/batch/cell line. The sigmoidal dose response curves compared to *F*-test and * indicates *p* < 0.05

In aEHT, 5-HT increased force at concentrations higher than 3 nM. Maximum effects were reached around 1 µM and the logEC_50_ value for 5-HT was estimated as −7.4 ± 0.3 M (*n *= 11/3/1, EHTs/batches/cell lines, Fig. 3). The potency of NE to increase force was very similar (logEC_50_: −7.0 ± 0.3 M; *n* = 9/3/1, EHTs/batches/cell lines), but E_max_ of NE was slightly larger than E_max_ of 5–HT (*F*-test, 0.12 ± 0.01 vs 0.10 ± 0.01 mN). A high extracellular Ca^2+^ concentration (1.8 mM) was used to estimate maximum force development. Values in the presence of 1.8 mM Ca^2+^ did not differ between aEHT pre-treated with NE or 5-HT: (0.16 ± 0.02 vs. 0.16 ± 0.01 mN, Fig. 3).

In vEHT, basal force was much larger than in aEHT (0.15 ± 0.01 vs 0.06 ± 0.01 mN). In contrast to aEHT, 5-HT did not evoke consistent inotropic effects in vEHT (Fig. 3). NE increased force with a logEC_50_ of −6.5 ± 0.01 M (E_max_ of NE: 0.17 ± 0.01 mN, 12/3/1). Once more, the force values in the presence of 1.8 mM Ca^2+^ did not differ between EHTs that had been pre-treated with 5-HT or NE (Fig. 3). In some experiments, we applied NE on top of 5-HT. NE increased force even in EHTs that did not show a positive inotropic effect upon 5-HT, indicating proper cAMP/PKA signaling.

The results were confirmed with EHTs from a second cell line (ERC018). Since basal forces differed from those in ERC001, data are presented in a separate figure (Figure S4, S5).

### 5-HT increased spontaneous beating rate in both atrial EHT and ventricular EHT

**Fig. 4:**
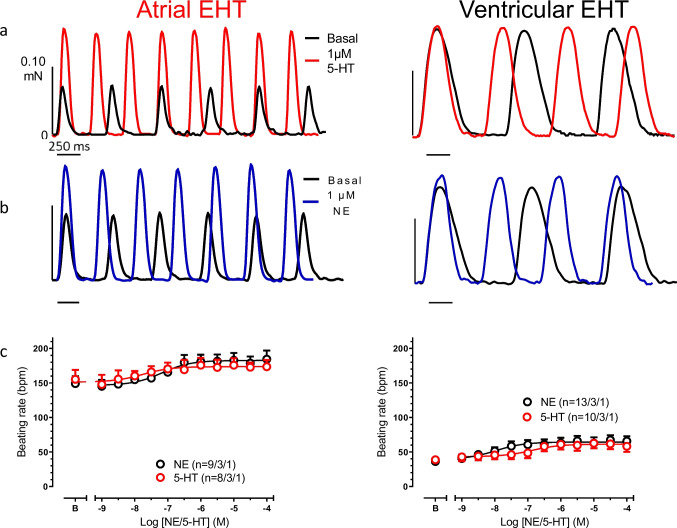
**5-HT increased spontaneous beating rate in atrial and ventricular EHT**. Respective time courses of contraction basal and at the 1 µM 5-HT (**a**) or NE (**b**) in atrial (left) and ventricular (right) EHT. Data set of spontaneous beating rate at the increasing 5-HT and NE concentrations (from 10^−9^ to 10^−4^ M) in atrial (left) and ventricular (right) EHT (**c**). Basal indicates as “**B”** at 0.6 mM Ca^2+^ and the increasing 5-HT and NE concentrations (from 10^−9^ to 10^−4^ M). Mean ± SEM, number indicates number of EHT/batch/cell line

5-HT increases funny current (I_f_) in human atrial myocytes via 5-HT_4_ receptors (Pino et al. 1998). From these results, one would expect a positive chronotropic effect of 5-HT on aEHT. Therefore, we analyzed the effect of 5-HT on beating frequency in both atrial and ventricular EHT. Again, NE was used for comparison. Basal beating rate was not different between aEHT exposed to 5-HT or NE. Both 5-HT and NE increased beating rate (Fig. 4). The effect size of NE or 5-HT on beating rate did not differ (delta: 42.9. ± 8.5 vs. 36.8 ± 10.5 beating per minute (bpm); 9/3/1 and 8/3/1). The potency of 5-HT was significantly higher than that of NE to increase beating rate in aEHT (log EC_50_ of NE vs. 5-HT: −7.1 ± 0.4 vs. −8.0 ± 0.5 M).

Surprisingly, beating rate in vEHT was not only increased by NE but also by 5-HT (Fig. 4). The potency of 5-HT was smaller than that of NE (log EC_50_ of NE vs. 5-HT: −7.9 ± 0.3 vs. −7.2 ± 0.3 M). Nevertheless, the effect size of NE or 5-HT on beating rate was not different (delta: 29.5 ± 3.1 vs. 25.0 ± 4.7 bpm, 13/3/1 and 10/3/1).

We also confirmed the chronotropic responses to 5-HT in EHTs from a second cell line (ERC018). Since basal spontaneous beating rate differed from those in ERC001, data are presented in a separate figure (Figure S4, 5).

### Effects of 5-HT on APD in vEHT

**Fig. 5:**
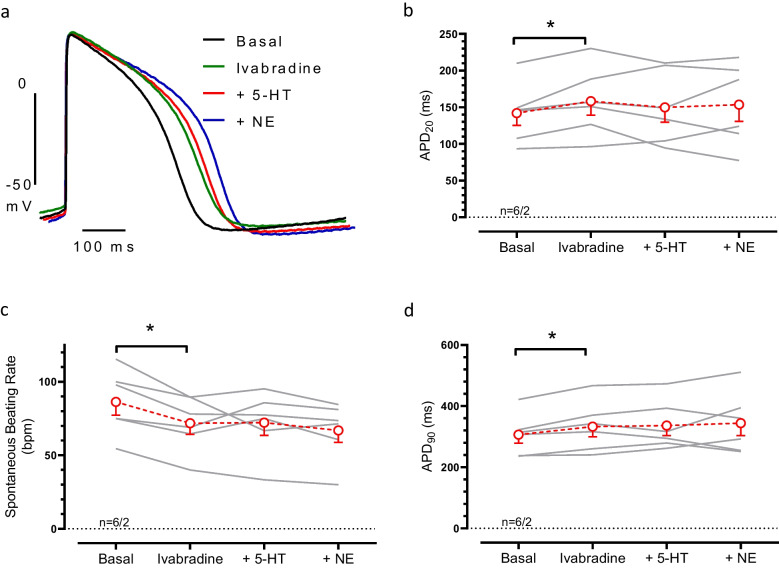
**The positive chronotropic effect of 5-HT in vEHT was abolished by I**_**f**_ **blockade**. Original action potential (AP) traces and data set of AP duration. AP recorded before (basal) and after adding 300 nM ivabradine, 100 µM 5-HT and 100 µM NE (**a**). Summary of action potential duration at % 20 repolarization (APD20, **b**), spontaneous beating rate (**c**) or action potential duration at % 90 repolarization (APD90, **d**) values. Mean ± SEM. p < 0.05, one-way ANOVA, n/n indicates number of EHT/batch

In contractility experiments, 5-HT increased beating rate in vEHT. In order to elucidate whether positive chronotropic effect of 5-HT in vEHT depends on activation of I_f_, we measured 5-HT effects in the presence of the I_f_ blocker ivabradine (300 nM). Ivabradine, significantly reduced the beating rate by ~ 17% (from 86.3 ± 9.0 to 71.8 ± 7.6 *n* = 6) and prolonged APD slightly. More importantly, positive chronotropic effects of both 5-HT and NE were abolished. In addition, 5-HT and NE did not show any significantly effect on APD (Fig. 5).

## Discussion

In this study, we demonstrate that aEHT recapitulate a finding exclusively described for the human atrium: direct positive inotropic effects of 5-HT. In line, we also found an increase in I_Ca_ and a prolongation of AP plateau phase.

### Positive inotropic effects of 5-HT in general

Positive inotropic effects of 5-HT are reported for several mammalian species (Neumann et al. 2023) and were initially interpreted as indirect effects, because 5-HT releases NE from intracardiac nerve endings (Trendelenburg 1960). However, in two species, 5 HT is able to evoke a direct inotropic effect: humans and pigs (Kaumann 1991). Positive inotropic effects are restricted to the atrium. Ventricular positive inotropic responses of 5-HT are restricted to newborn pigs when PDE3 and PDE4 are inhibited together (Galindo-Tovar et al. 2009). No data from humans are available. Here, we report positive inotropic effects paralleled by an increases in I_Ca_ in hiPSC-CM. Thus, we feel confident that the effects we described in that study relate to direct activation of 5-HT_4_ receptors.

### Both hiPSC-aCM and hiPSC-vCM express 5-HT_4_ receptors

In the human heart, inotropic effects of 5-HT are restricted to the atrium. However, there is no doubt that ventricular tissue also expresses a substantial number of 5-HT_4_ receptors. The mRNA abundance of genes encoding for 5-HT_4_ receptors in human ventricle was about 40% of that in the atrium (Gergs et al. 2009). Our EHT model nicely recapitulates this principal finding of a higher *HTR4* in atrial vs. ventricular tissue. The atrial dominant expression for *HTR4* was even larger than in the mentioned above study in human tissue. In the past, atrial tissue from mice expressing human 5-HT4 receptors was used to measure acute desensitization of G-protein coupled receptors (Gergs et al. 2017). Since EHT can be cultivated over weeks, they should also enable investigation of chronic effects of receptor activation.

### 5-HT effects on I_Ca_

5-HT increases I_Ca_ in human atrial CM (Jahnel et al. 1992; Ouadid et al. 1992), but not in human ventricular CM (Uzun et al. 2016). This finding is nicely reflected in our hiPSC-CM model. There are substantial quantitative differences between hiPSC-aCM and adult atrial CM. In hiPSC-aCM, I_Ca_ increase by 5-HT is less than 50% of that described for human atrial CM (Berk et al. 2016; Christ et al. 2014). This finding does not imply per se weak 5-HT effects in hiPSC-aCM, since NE effects in hiPSC-aCM are also smaller than in adult human atrial CM. The same holds true for the effects of 5-HT on plateau phase of AP and on force in aEHT. The weaker 5-HT effects cannot be explained by a lower potency of 5-HT, since logEC_50_ for the positive inotropic effect in aEHT was even slightly higher than for NE (7.5 vs. 7.0). Obviously, maximum increases in I_Ca_ in hiPSC-CM are generally smaller than in adult human CM as previously shown (Uzun et al. 2016). PDEs are not involved in that peculiarity (Iqbal et al. 2021). Thus, the reason for these general differences remains open.

### Chronotropic effect of 5-HT in vEHT: I_f_ activation in the absence of I_Ca_ activation

In human atrial CM, β-adrenoceptor but also 5-HT_4_ receptor activation increases not only I_Ca_ (Berk et al. 2016) but also I_f_ (Pino et al. 1998). In aEHT (this study), NE and 5-HT increased the beating rate to the same extent, while the increases in I_Ca_, plateau duration, and force were significantly smaller with 5-HT than with NE. Thus, one could speculate that activation of I_f_ in aEHT (by 5-HT or NE) may be more important for the positive chronotropic effects described in aEHT. In vEHT, the discrepancy between 5-HT action on beating rate (as large as in NE) and on I_Ca_ (no effect) is even more pronounced. It is well described that I_f_ can be activated directly by cAMP without the involvement of PKA (DiFrancesco and Tortora 1967). The positive chronotropic effect of 5-HT in vEHT is in line with expression of 5-HT receptors in human ventricular tissue. Obviously, chronotropic response of 5-HT on vEHT depends on I_f_ because 5-HT do not increase I_Ca_ in hiPSC-CM and chronotropic effects are completely blunted by I_f_ block. As reported before for human adult ventricular tissue, neither 5-HT (Jahnel et al. 1992) nor NE (Lemoine et al. 2018) had an effect of APD in our vEHT. Thus, we speculate that in hiPSC-CM, 5-HT receptors might activate the production of cAMP without access to a pool that is able to activate PKA.

In our aEHT experiments, 5-HT increased APD_20_ as reported before by Pau et al. However, effects on APD_90_ differed fundamentally. APD_90_ prolonged in isolated atrial CM, but shortened in adult atrial tissue and in aEHT. We expect methodological issues. AP plateau voltage is very low in human atrium and any shift to more positive voltages will increase voltage-dependent activation of hERG channels mediating final repolarization. As a result, the effect of lifting up the plateau voltage by blocking will critically depend on I_Kr_; There is a shortening under physiological conditions but a prolongation when I_Kr_ is blocked (Wettwer et al. 2004). Since hERG channels are very prone to enzymatic damage by serine protease used for cell isolation, we expect that the prolongation of APD_90_ with 5-HT seen in isolated atrial CM may result from an impairment of I_Kr_. For a detailed discussion, see also Schulz et al. (2024).

In conclusion, atrial EHT responded to 5-HT with a positive inotropic and chronotropic effect, aligning with observations in the human atrium. This response originates from an increased calcium current in hiPSC-aCM, similar to the mechanism found in human aCM.

## Limitations

As shown with the AP shape, the efficacy of RA to induce an atrial phenotype may vary substantially (Schulz, et al. 2024). Thus, one could speculate that responses on I_Ca_ by 5-HT lower than NE responses may indicate still imperfect “atrialization” on hiPSC-CM.

We have not measured effects of PDE on 5-HT in our aEHT. From work in vEHT, we expect a huge impact of PDE on the regulation of β-adrenoceptor-mediated effects (Iqbal et al. 2021; Saleem et al. 2020).

We have shown only data on mRNA abundance for HTR4. Comparison of the abundance of the other constituents of the HTR4 signalosome in EHT vs. adult heart tissue is highly desirable to get deeper insights, but was felt beyond the scope of this study.

## Supplementary Information

Below is the link to the electronic supplementary material.

ESM 1 (PPTX 140 KB)

## Data Availability

All source data for this work (or generated in this study) are available upon reasonable request.

## References

[CR1] Berk E, Christ T, Schwarz S, Ravens U, Knaut M, Kaumann AJ (2016) In permanent atrial fibrillation, PDE3 reduces force responses to 5-HT, but PDE3 and PDE4 do not cause the blunting of atrial arrhythmias. Br J Pharmacol 173(16):2478–2489. 10.1111/bph.13525 27238373 10.1111/bph.13525 PMC4959958

[CR2] Breckwoldt K, Letuffe-Brenière D, Mannhardt I, Schulze T, Ulmer B, Werner T, Benzin A, Klampe B, Reinsch MC, Laufer S, Shibamiya A, Prondzynski M, Mearini G, Schade D, Fuchs S, Neuber C, Krämer E, Saleem U, Schulze ML, Rodriguez ML, Eschenhagen T, Hansen A (2017) Differentiation of cardiomyocytes and generation of human engineered heart tissue. Nat Protoc 12(6):1177–1197. 10.1038/nprot.2017.033 28492526 10.1038/nprot.2017.033

[CR3] Christ T, Rozmaritsa N, Engel A, Berk E, Knaut M, Metzner K, Canteras M, Ravens U, Kaumann A (2014) Arrhythmias, elicited by catecholamines and serotonin, vanish in human chronic atrial fibrillation. Proc Natl Acad Sci U S A 111(30):11193–11198. 10.1073/pnas.1324132111 25024212 10.1073/pnas.1324132111 PMC4121801

[CR4] Devalla, Harsha D., Verena Schwach, John W. Ford, James T. Milnes, Said El‐Haou, Claire Jackson, Konstantinos Gkatzis, David A. Elliott, Susana M. Chuva de Sousa Lopes, Christine L. Mummery, Arie O. Verkerk, and Robert Passier. 2015. “Atrial‐like cardiomyocytes from human pluripotent stem cells are a robust preclinical model for assessing atrial‐selective pharmacology.” *EMBO Molecular Medicine* 7(4):394–410. 10.15252/emmm.201404757. 10.15252/emmm.201404757 PMC4403042 25700171

[CR5] DiFrancesco D, Tortora P (1967) Direct activation of cardiac pacemaker channels by intracellular cyclic AMP. Nature 27(18):845–855 10.1038/351145a0 1709448

[CR6] Dolce B, Christ T, Pavlidou NG, Yildirim Y, Reichenspurner H, Eschenhagen T, Nikolaev VO, Kaumann AJ, Molina CE (2020) Impact of phosphodiesterases PDE3 and PDE4 on 5-hydroxytryptamine receptor4-mediated increase of CAMP in human atrial fibrillation. Naunyn-Schmiedebergs Arch Pharmacol. 10.1007/s00210-020-01968-1 32949251 10.1007/s00210-020-01968-1 PMC7835186

[CR7] Ellinghaus P, Scheubel RJ, Dobrev D, Ravens U, Holtz J, Huetter J, Nielsch U, Morawietz H (2005) Comparing the global MRNA expression profile of human atrial and ventricular myocardium with high-density oligonucleotide arrays. J Thorac Cardiovasc Surg 129(6):1383–1390. 10.1016/j.jtcvs.2004.08.031 15942582 10.1016/j.jtcvs.2004.08.031

[CR8] Gaborit N, Le Bouter S, Szuts V, Varro A, Escande D, Nattel S, Demolombe S (2007) Regional and tissue specific transcript signatures of ion channel genes in the non-diseased human heart. J Physiol 582(2):675–693. 10.1113/jphysiol.2006.126714 17478540 10.1113/jphysiol.2006.126714 PMC2075332

[CR9] Galindo-Tovar A, Vargas ML, Escudero E, Kaumann AJ (2009) Ontogenic changes of the control by phosphodiesterase-3 and -4 of 5-HT responses in porcine heart and relevance to human atrial 5-HT 4 receptors. Br J Pharmacol 156(2):237–249. 10.1111/j.1476-5381.2008.00023.x 19154438 10.1111/j.1476-5381.2008.00023.x PMC2697829

[CR10] Gergs U, Neumann J, Simm A, Silber RE, Remmers FO, Läer S (2009) Phosphorylation of phospholamban and troponin I through 5-HT4 receptors in the isolated human atrium. Naunyn-Schmiedebergs Arch Pharmacol 379(4):349–359. 10.1007/s00210-008-0371-y 19002436 10.1007/s00210-008-0371-y

[CR11] Gergs U, Fritsche J, Fabian S, Christ J, Neumann J (2017) Desensitization of the human 5-HT4 receptor in isolated atria of transgenic mice. Naunyn-Schmiedebergs Arch Pharmacol 390(10):987–996. 10.1007/s00210-017-1403-2 28689254 10.1007/s00210-017-1403-2

[CR12] Hansen A, Eder A, Bonstrup M, Flato M, Mewe M, Schaaf S, Aksehirlioglu B, Schworer A, Uebeler J, Eschenhagen T, Eschenhagen T (2010) Development of a drug screening platform based on engineered heart tissue. Circ Res. 10.1161/CIRCRESAHA.109.211458 20448218 10.1161/CIRCRESAHA.109.211458

[CR13] Iqbal Z, Ismaili D, Dolce B, Petersen J, Reichenspurner H, Hansen A, Kirchhof P, Eschenhagen T, Nikolaev VO, Molina CE, Christ T (2021) Regulation of basal and norepinephrine-induced CAMP and ICa in HiPSC-cardiomyocytes: effects of culture conditions and comparison to adult human atrial cardiomyocytes. Cell Signal 82(January):109970. 10.1016/j.cellsig.2021.109970 33677066 10.1016/j.cellsig.2021.109970

[CR14] Jahnel U, Rupp J, Ertl R, Nawrath H (1992) Positive inotropic response to 5-HT in human atrial but not in ventricular heart muscle. J Pharmacol Exp Ther 2:482–485 10.1007/BF00169000 1335123

[CR15] Kaumann AJ (1991) 5-HT4-like receptors in mammalian atria. J Neural Transm Suppl 34:195-201. 10.1007/978-3-7091-9175-0_25 10.1007/978-3-7091-9175-0_25 1667872

[CR16] Kaumann AJ, Sanders L (1994) 5-hydroxytryptamine causes rate-dependent arrhythmias through 5-HT4 receptors in human atrium: facilitation by chronic β-adrenoceptor blockade. Naunyn-Schmiedebergs Arch Pharmacol 349(4):331–337. 10.1007/BF00170877 7914677 10.1007/BF00170877

[CR17] Kaumann AJ, Sanders L, Brown AM, Murray KJ, Brown MJ (1990) A 5-hydroxytryptamine receptor in human atrium. Br J Pharmacol 100(4):879–885. 10.1111/j.1476-5381.1990.tb14108.x 2169944 10.1111/j.1476-5381.1990.tb14108.x PMC1917575

[CR18] Lee JH, Protze SI, Laksman Z, Backx PH, Keller GM (2017) Human pluripotent stem cell-derived atrial and ventricular cardiomyocytes develop from distinct mesoderm populations. Cell Stem Cell 21(2):179-194.e4. 10.1016/j.stem.2017.07.003 28777944 10.1016/j.stem.2017.07.003

[CR19] Lemme M, Ulmer BM, Lemoine MD, Zech ATL, Flenner F, Ravens U, Reichenspurner H, Rol-Garcia M, Smith G, Hansen A, Christ T, Eschenhagen T (2018) Atrial-like engineered heart tissue: an in vitro model of the human atrium. Stem Cell Reports 11(6):1378–1390. 10.1016/j.stemcr.2018.10.008 30416051 10.1016/j.stemcr.2018.10.008 PMC6294072

[CR20] Lemoine MD, Mannhardt I, Breckwoldt K, Prondzynski M, Flenner F, Ulmer B, Hirt MNMN, Neuber C, Horváth A, Kloth B, Reichenspurner H, Willems S, Hansen A, Eschenhagen T, Christ T (2017) Human IPSC-derived cardiomyocytes cultured in 3d engineered heart tissue show physiological upstroke velocity and sodium current density. Sci Rep. 10.1038/s41598-017-05600-w 28710467 10.1038/s41598-017-05600-w PMC5511281

[CR21] Lemoine MD, Krause T, Koivumäki JT, Prondzynski M, Schulze ML, Girdauskas E, Willems S, Hansen A, Eschenhagen T, Christ T (2018) Human induced pluripotent stem cell-derived engineered heart tissue as a sensitive test system for QT prolongation and arrhythmic triggers. Circ Arrhythm Electrophysiol. 10.1161/CIRCEP.117.006035 29925535 10.1161/CIRCEP.117.006035

[CR22] Mannhardt I, Eder A, Dumotier B, Prondzynski M, Kr-amer E, Traebert M, Flenner F, Stathopoulou K, Lemoine MD, Carrier L, Christ T, Eschenhagen T, Hansen A (2017) Blinded contractility analysis in Hipsc-cardiomyocytes in engineered heart tissue format: comparison with human atrial trabeculae. Toxicol Sci 158(1):164–175. 10.1093/toxsci/kfx081 28453742 10.1093/toxsci/kfx081 PMC5837217

[CR23] Neumann J, Hofmann B, Dhein S, Gergs U (2023) Cardiac roles of serotonin (5-HT) and 5-HT-receptors in health and disease. Int J Mol Sci. 10.3390/ijms24054765 36902195 10.3390/ijms24054765 PMC10003731

[CR24] Ouadid H, Seguin J, Dumuis A, Bockaert J, Nargeot J (1992) Serotonin increases calcium current in human atrial myocytes via the newly described 5-hydroxytryptamine4 receptors. Mol Pharmacol 41(2):346–351 1311410

[CR25] Pau D, Workman AJ, Kane KA, Rankin AC (2007) Electrophysiological and arrhythmogenic effects of 5-hydroxytryptamine on human atrial cells are reduced in atrial fibrillation. J Mol Cell Cardiol 42(1):54–62. 10.1016/j.yjmcc.2006.08.007 16989857 10.1016/j.yjmcc.2006.08.007 PMC2526346

[CR26] Pecha S, Ismaili D, Geelhoed B, Knaut M, Reichenspurner H, Eschenhagen T, Schnabel RB, Christ T, Ravens U (2023) Resting membrane potential is less negative in trabeculae from right atrial appendages of women, but action potential duration does not shorten with age. J Mol Cell Cardiol 176(January):1–10. 10.1016/j.yjmcc.2023.01.006 36681268 10.1016/j.yjmcc.2023.01.006

[CR27] Pino R, Cerbai E, Calamai G, Alajmo F, Borgioli A, Braconi L, Cassai M, Montesi GF, Mugelli A (1998) Effect of 5-HT4 receptor stimulation on the pacemaker current I(f) in human isolated atrial myocytes. Cardiovasc Res 40(3):516–522. 10.1016/S0008-6363(98)00198-9 10070492 10.1016/s0008-6363(98)00198-9

[CR28] Roselli, Carolina, Ida Surakka, Morten S. Olesen, Gardar Sveinbjornsson, Nicholas A. Marston, Seung Hoan Choi, Hilma Holm, Mark Chaffin, Daniel Gudbjartsson, Matthew C. Hill, Hildur Aegisdottir, Christine M. Albert, Alvaro Alonso, Christopher D. Anderson, Dan E. Arking, David O. Arnar, John Barnard, Emelia J. Benjamin, Eugene Braunwald, Ben Brumpton, Archie Campbell, Nathalie Chami, Daniel I. Chasman, Kelly Cho, Eue-Keun Choi, Ingrid E. Christophersen, Mina K. Chung, David Conen, Harry J. Crijns, Michael J. Cutler, Tomasz Czuba, Scott M. Damrauer, Martin Dichgans, Marcus Dörr, Elton Dudink, ThuyVy Duong, Christian Erikstrup, Tõnu Esko, Diane Fatkin, Jessica D. Faul, Manuel Ferreira, Daniel F. Freitag, Santhi K. Ganesh, J. Michael Gaziano, Bastiaan Geelhoed, Jonas Ghouse, Christian Gieger, Franco Giulianini, Sarah E. Graham, Vilmundur Gudnason, Xiuqing Guo, Christopher Haggerty, Caroline Hayward, Susan R. Heckbert, Kristian Hveem, Kaoru Ito, Renee Johnson, J. Wouter Jukema, Sean J. Jurgens, Stefan Kääb, John P. Kane, Shinwan Kany, Sharon L. R. Kardia, Maryam Kavousi, Shaan Khurshid, Frederick K. Kamanu, Paulus Kirchhof, Marcus E. Kleber, Stacey Knight, Issei Komuro, Jose E. Krieger, Lenore J. Launer, Dadong Li, Honghuang Lin, Henry J. Lin, Ruth J. F. Loos, Luca Lotta, Steven A. Lubitz, Kathryn L. Lunetta, Peter W. Macfarlane, Patrik K. E. Magnusson, Rainer Malik, Helene Mantineo, Gregory M. Marcus, Winfried März, David D. McManus, Olle Melander, Giorgio E. M. Melloni, Pascal B. Meyre, Kazuo Miyazawa, Sanghamitra Mohanty, Laia M. Monfort, Martina Müller-Nurasyid, Navid A. Nafissi, Andrea Natale, Saman Nazarian, Sisse R. Ostrowski, Hui-Nam Pak, Shichao Pang, Ole B. Pedersen, Nancy L. Pedersen, Alexandre C. Pereira, James P. Pirruccello, Michael Preuss, Bruce M. Psaty, Clive R. Pullinger, Daniel J. Rader, Joel T. Rämö, Paul M. Ridker, Michiel Rienstra, Lorenz Risch, Dan M. Roden, Jerome I. Rotter, Marc S. Sabatine, Heribert Schunkert, Svati H. Shah, Jaemin Shim, M. Benjamin Shoemaker, Bridget Simonson, Moritz F. Sinner, Roelof A. J. Smit, Jennifer A. Smith, Nicholas L. Smith, J. Gustav Smith, Elsayed Z. Soliman, Erik Sørensen, Nona Sotoodehnia, Daniel Strbian, Bruno H. Stricker, Maris Teder-Laving, Yan V Sun, Sébastien Thériault, Rosa B. Thorolfsdottir, Unnur Thorsteinsdottir, Arnljot Tveit, Pim van der Harst, Joyce van Meurs, Biqi Wang, Stefan Weiss, Quinn S. Wells, Lu-Chen Weng, Peter W. Wilson, Ling Xiao, Pil-Sung Yang, Jie Yao, Zachary T. Yoneda, Tanja Zeller, Lingyao Zeng, Wei Zhao, Xiang Zhou, Sebastian Zöllner, BioBank Japan Project, Regeneron Genetics Center, DBDS Genomic Consortium, Christian T. Ruff, Henning Bundgaard, Cristen Willer, Kari Stefansson, and Patrick T. Ellinor (2025) Meta-analysis of genome-wide associations and polygenic risk prediction for atrial fibrillation in more than 180,000 cases. Nat Genet 57(3):539–547. 10.1038/s41588-024-02072-3 40050429 10.1038/s41588-024-02072-3 PMC12094172

[CR29] Saleem U, Ismaili D, Mannhardt I, Pinnschmidt H, Schulze T, Christ T, Eschenhagen T, Hansen A (2020) Regulation of ICa,L and force by PDEs in human-induced pluripotent stem cell-derived cardiomyocytes. Br J Pharmacol 177(13):3036–3045. 10.1111/bph.15032 32092149 10.1111/bph.15032 PMC7279982

[CR30] Schulz C, Sönmez M, Krause J, Schwedhelm E, Bangfen P, Alihodzic D, Hansen A, Eschenhagen T, Christ T (2023) A critical role of retinoic acid concentration for the induction of a fully human-like atrial action potential phenotype in HiPSC-CM. Stem Cell Rep 18(11):2096–2107. 10.1016/j.stemcr.2023.10.006 10.1016/j.stemcr.2023.10.006 PMC10679650 37922915

[CR31] Schulz C, Eschenhagen T, Christ T (2024) Atrial HiPSC-CM as a pharmacological model to evaluate anti-AF drugs: some lessons from IKur. J Cardiovasc Pharmacol 84(5):479–485. 10.1097/FJC.0000000000001631 39270001 10.1097/FJC.0000000000001631

[CR32] Trautwein W, Kassebaum DG, Nelson RM, Hecht HH (1962) Electrophysiological study of human heart muscle. Circ Res 10:306–312. 10.1161/01.RES.10.3.306 13922328 10.1161/01.res.10.3.306

[CR33] Trendelenburg U (1960) The action of histamine and 5-hydroxytryptamine on isolated mammalian atria. J Pharmacol Exp Ther 130:450 13777992

[CR34] Uzun, Ahmet U., Ingra Mannhardt, Kaja Breckwoldt, András Horváth, Silke S. Johannsen, Arne Hansen, Thomas Eschenhagen, and Torsten Christ. 2016. “Ca2+-currents in human induced pluripotent stem cell-derived cardiomyocytes effects of two different culture conditions.” *Frontiers in Pharmacology* 7(SEP). 10.3389/fphar.2016.00300. 10.3389/fphar.2016.00300 PMC5018497 27672365

[CR35] Wettwer E, Hála O, Christ T, Heubach JF, Dobrev D, Knaut M, Varró A, Ravens U (2004) Role of IKur in controlling action potential shape and contractility in the human atrium: influence of chronic atrial fibrillation. Circulation 110(16):2299–2306. 10.1161/01.CIR.0000145155.60288.71 15477405 10.1161/01.CIR.0000145155.60288.71

