# Supplementary material for: Positive inotropic effects of serotonin in atrial EHT: further proof for an atrial phenotype?
Source: Naunyn Schmiedebergs Arch Pharmacol. 2025 Oct 28;399(4):5055–66. doi: 10.1007/s00210-025-04619-5 (PMC13046593; doi:10.1007/s00210-025-04619-5)
Supplement: Supplementary file 1 — (PPTX 140 KB) [file 210_2025_4619_MOESM1_ESM.pptx]

## Slide 1
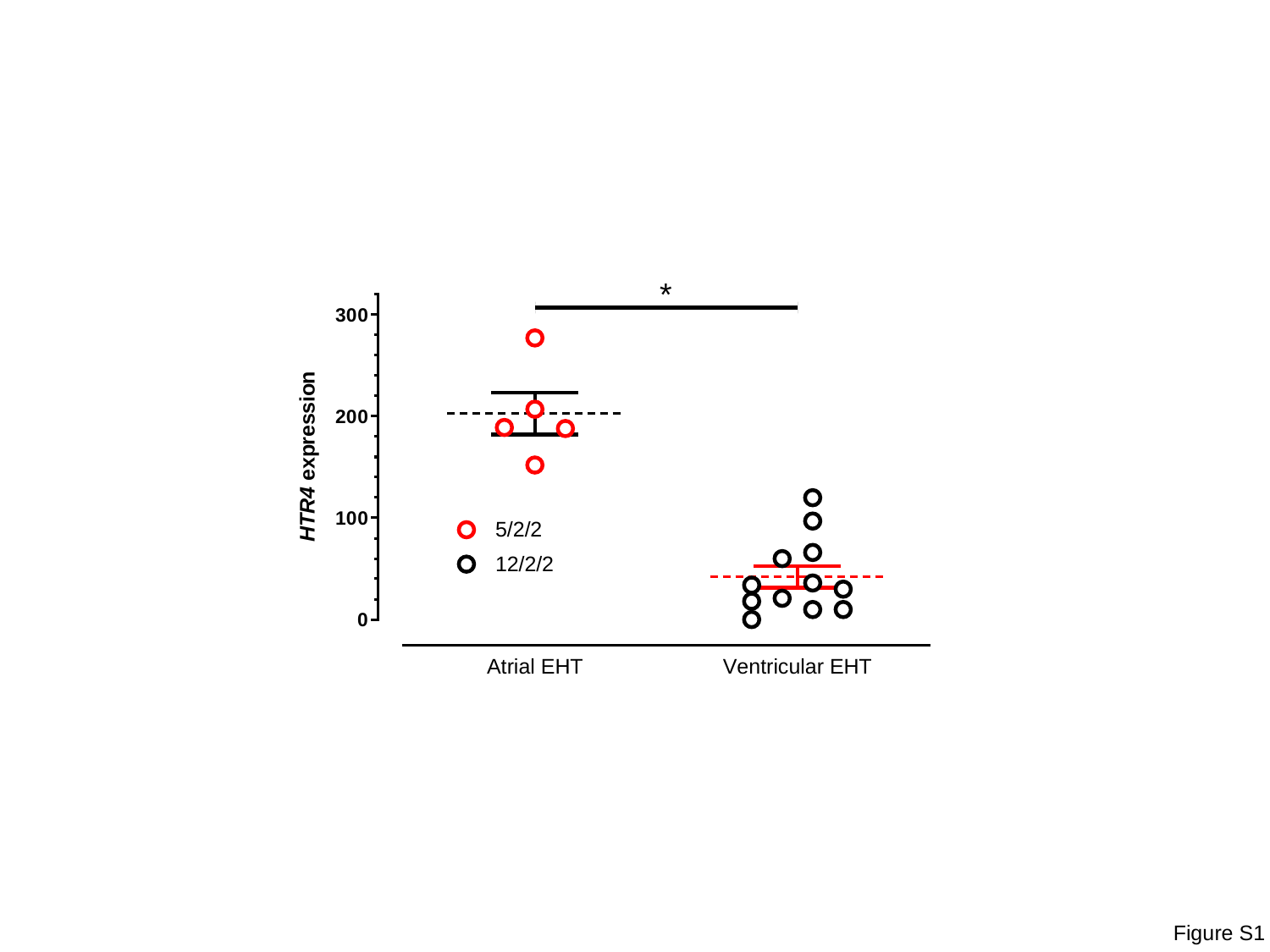

Figure S1

## Slide 2
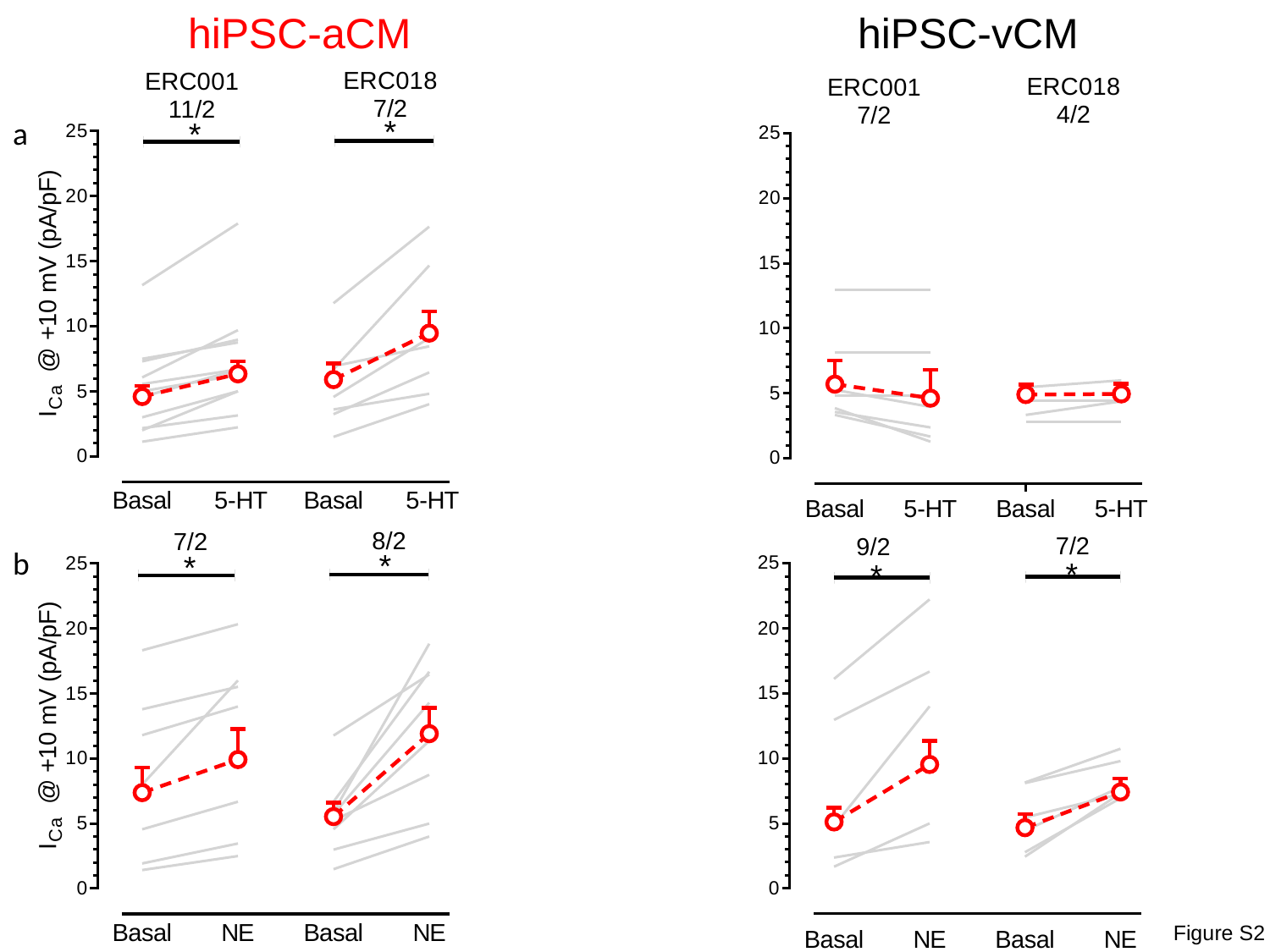

hiPSC-aCM
hiPSC-vCM
a
b
Figure S2

## Slide 3
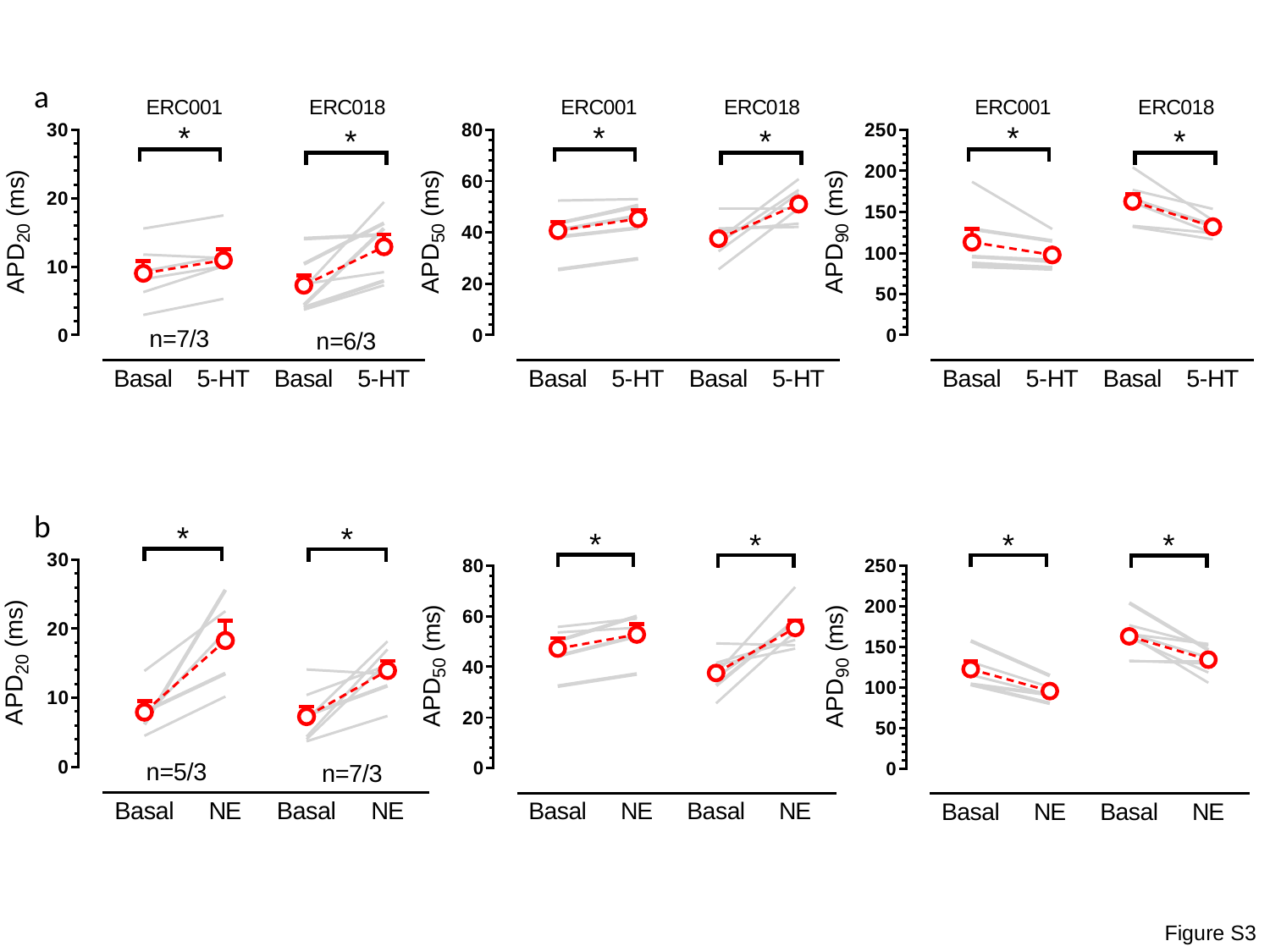

a
b
Figure S3

## Slide 4
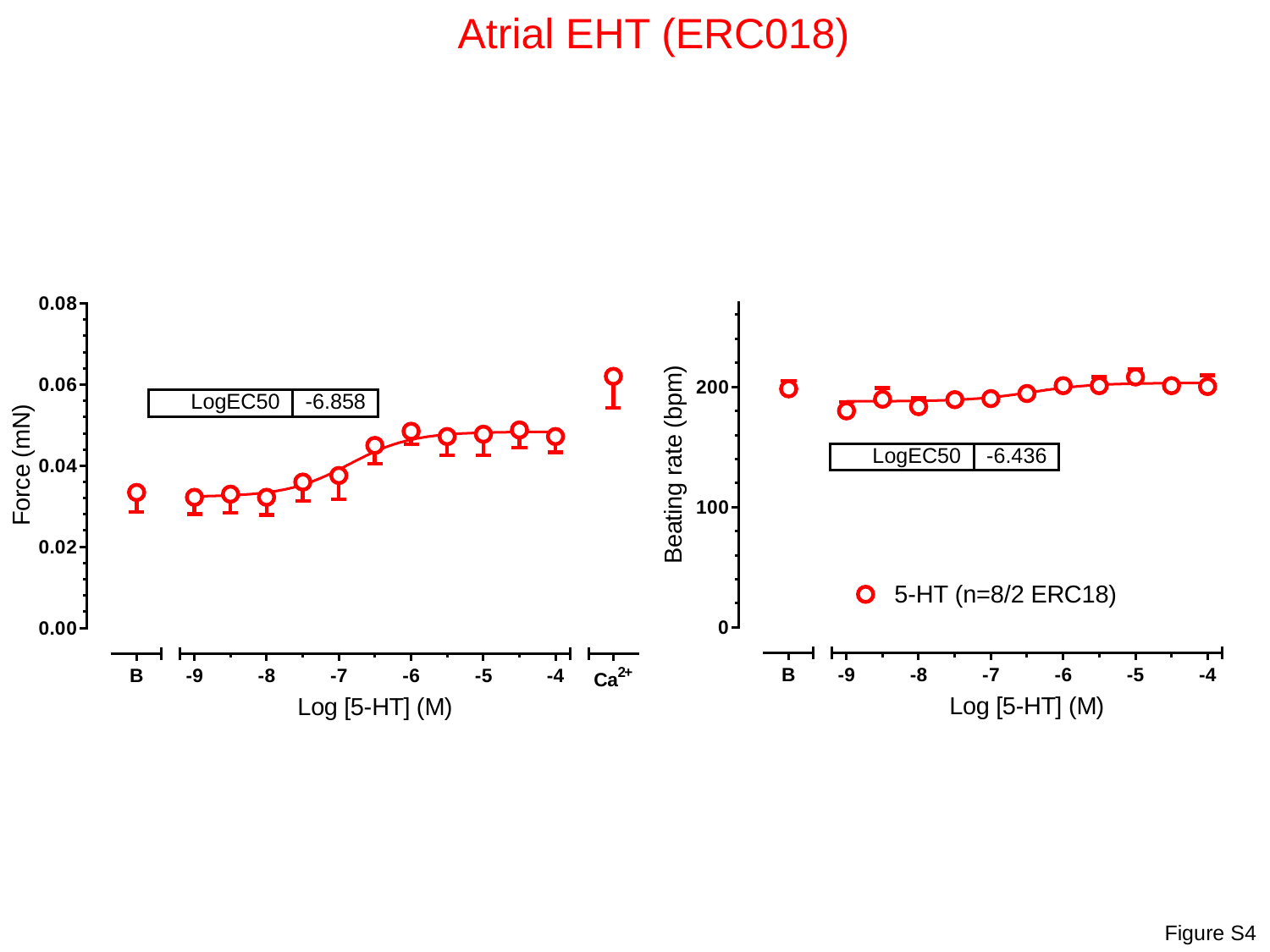

Atrial EHT (ERC018)
Figure S4

## Slide 5
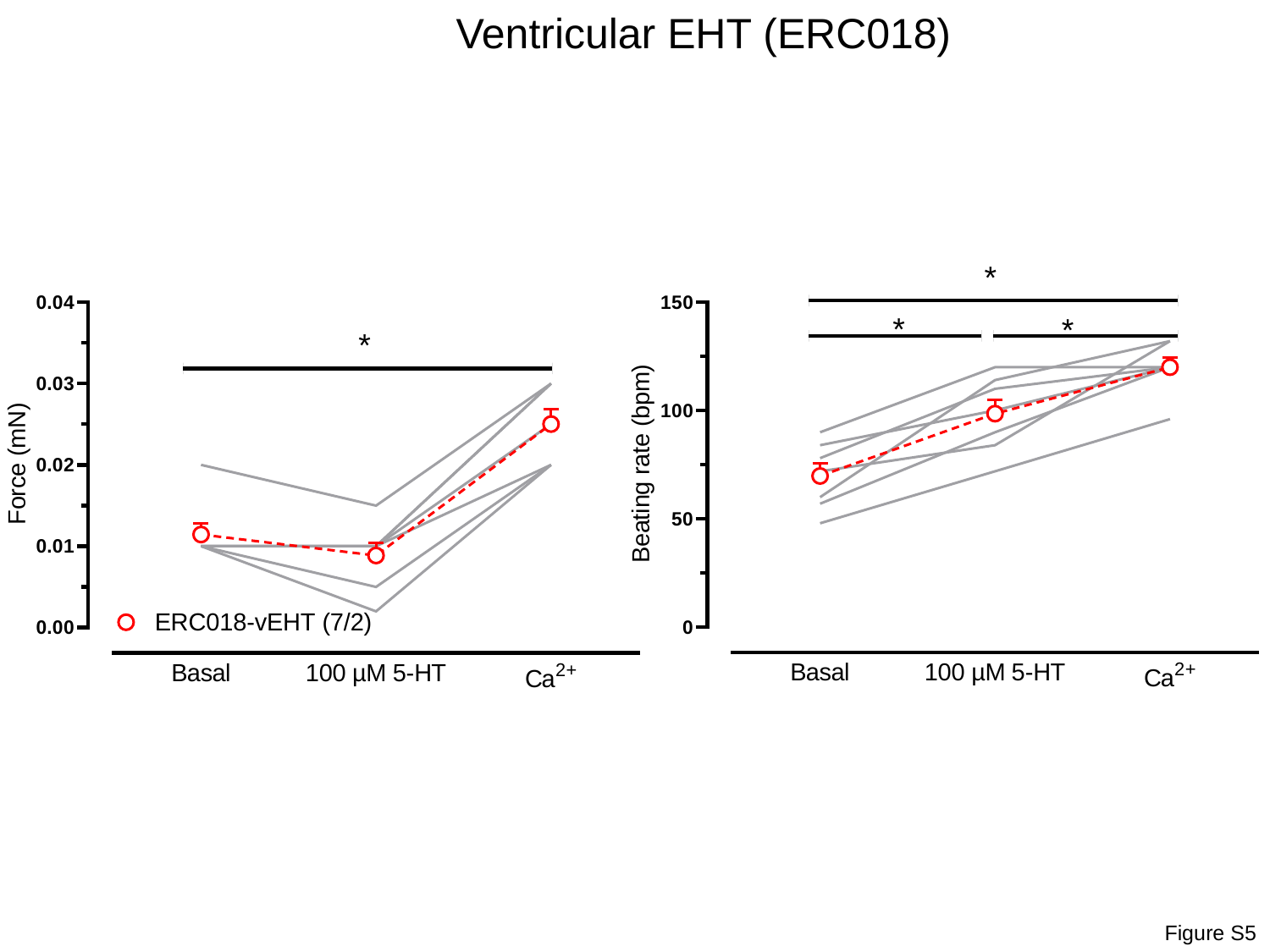

Ventricular EHT (ERC018)
Figure S5
